# Simulation and Modelling of C+L+S Multiband Optical Transmission for the OCATA Time Domain Digital Twin †

**DOI:** 10.3390/s25061948

**Published:** 2025-03-20

**Authors:** Prasunika Khare, Nelson Costa, Marc Ruiz, Antonio Napoli, Jaume Comellas, Joao Pedro, Luis Velasco

**Affiliations:** 1 Advanced Broadband Communications Center (CCABA), Universitat Politècnica de Catalunya (UPC), 08034 Barcelona, Spain; prasunika.khare@upc.edu (P.K.); marc.ruiz-ramirez@upc.edu (M.R.); jaume.comellas@upc.edu (J.C.); 2 Infinera Unipessoal Lda., 2790-078 Carnaxide, Portugal; ncosta@infinera.com (N.C.); jpedro@infinera.com (J.P.); 3 Infinera, 81541 Munich, Germany; anapoli@infinera.com; 4 Instituto de Telecomunicações, Instituto Superior Técnico, 1049-001 Lisbon, Portugal

**Keywords:** multiband optical transmission, signal propagation modelling, digital twin modelling

## Abstract

C+L+S multiband (MB) optical transmission has the potential to increase the capacity of optical transport networks, and thus, it is a possible solution to cope with the traffic increase expected in the years to come. However, the introduction of MB optical technology needs to come together with the needed tools that support network planning and operation. In particular, quality of transmission (QoT) estimation is needed for provisioning optical MB connections. In this paper, we concentrate on modelling MB optical transmission for provide fast and accurate QoT estimation and propose machine learning (ML) approaches based on neural networks, which can be easily integrated into an optical layer digital twin (DT) solution. We start by considering approaches that can be used for accurate signal propagation modelling. Even though solutions such as the split-step Fourier method (SSFM) for solving the nonlinear Schrödinger equation (NLSE) have limited application for QoT estimation during provisioning because of their very high complexity and time consumption, they could be used to generate datasets for ML model creation. However, even that can be hard to carry out on a fully loaded MB system with hundreds of channels. In addition, in MB optical transmission, interchannel stimulated Raman scattering (ISRS) becomes a major effect, which adds more complexity. In view of that, the fourth-order Runge–Kutta in the interaction picture (RK4IP) method, complemented with an adaptive step size algorithm to further reduce the computation time, is evaluated as an alternative to reduce time complexity. We show that RK4IP provided an accuracy comparable to that of the SSFM with reduced computation time, which enables its application for MB optical transmission simulation. Once datasets were generated using the adaptive step size RK4IP method, two ML modelling approaches were considered to be integrated in the OCATA DT, where models predict optical signal propagation in the time domain. Being able to predict the optical signal in the time domain, as it will be received after propagation, opens opportunities for automating network operation, including connection provisioning and failure management. In this paper, we focus on comparing the proposed ML modelling approaches in terms of the models’ general and QoT estimation accuracy.

## 1. Introduction

Mobile traffic is expected to double in the coming years because of the massive deployment of beyond 5G and Internet of things (IoT) devices and services [1]. This explosion in the number of connected devices and traffic is expected to push optical network capacity beyond its current limit. In view of that, the study in [2] shows that multiband (MB) optical transmission would be required at all network segments, including metro-aggregation, metro-core, and backbone, by the end of this decade. MB optical systems expand the available capacity of optical fibers by enabling transmission within, e.g., the S-, C-, and L-bands, which increases the transmission capacity with respect to wavelength division multiplexing (WDM) transmission in the C-band only. As a result, MB optical systems can reduce the number of fibers needed, thus postponing the deployment of new fibers [3].

The deployment of MB optical networks requires the development of new tools for their planning and operation, including optical connection (lightpath) provisioning [4] and failure management [5]. The most basic functionality that is required is the estimation of the quality of transmission (QoT) of lightpaths, e.g., in terms of the signal-to-noise ratio (SNR), the pre-forward-error-correction (FEC) bit error rate (BER), or the Q-factor. Such QoT estimation functionality needs to provide accurate results and have low computational complexity so planning and operation can be carried out in a timely manner.

Different approaches can be found in the literature for estimation of the QoT of a lightpath, where the estimation of linear (LI) and nonlinear (NLI) impairments are considered. In particular, signal propagation methods for solving the nonlinear Schrödinger equation (NLSE), where the split-step Fourier method (SSFM) [6] can be used to obtain accurate QoT estimations. However, the SSFM method entails high computational complexity. Other approaches have been proposed to reduce the complexity of the SSFM, such as the fourth-order Runge–Kutta (RK) in the interaction picture (RK4IP) integration method [7,8]. However, both these methods, although very accurate, require high time, which discards its application for network operation.

Analytical models offer accurate QoT estimations with moderate computational complexity, as compared with the SSFM and RK4IP. E.g., the Gaussian noise (GN) model [9] typically requires a few minutes per WDM channel [10]. It is for this very reason that analytical models have been intensively investigated. However, such computation time might still be too long for network planning and operation, and closed-form approximations, which can provide QoT estimations in less time, have been also investigated. This is especially important in the case of MB optical transmission, where fully loaded MB systems consist of several hundreds of channels. In addition, accurate QoT estimation in MB optical transmission is much more computationally complex than in traditional C-band optical transmission, mainly because of the interchannel stimulated Raman scattering (ISRS) [11] effect. In this regard, the authors in [12] analyzed the computational time and accuracy of several QoT estimation methods suitable for MB optical transmission, including (i) the numerical generalized GN model; (ii) the closed-form ISRS-GN model, and (iii) an improved four-wave mixing (FWM) model that accounted also for the ISRS effect. They showed that the closed-form ISRS-GN model had the best trade-off between computational complexity (speed) and accuracy.

Machine learning (ML) techniques have been also investigated for QoT estimation, as they can provide both good accuracy and low complexity. ML modelling includes: (i) dataset population; (ii) training, validation, and testing for creating the models; and (iii) using the models. ML model creation can be carried out from existing datasets. Populating the datasets with the right data is the most time-consuming and most important task in the ML approach, where data can come from simulation, lab experiments, and/or telemetry data collected from already established lightpaths. The datasets are used for model creation, which is also carried out beforehand to create a model database. Once the models are made available, using them for prediction is a straightforward task, so QoT estimation can be done in a very time-efficient way. Several works can be found in the literature for QoT estimation using ML; e.g., the authors in [13] evaluated the effectiveness of using ML models for QoT estimation and showed that neural networks (NNs) were able to accurately predict the QoT while presenting good generalization. A comparative study of QoT estimation based on the GN model and NNs is presented in [14], where the authors showed that under perfect knowledge of system parameters, GN-based analytical models may outperform NN models. However, inaccurate knowledge of various link parameters, which is common in practice, degrades GN-based models, and NNs generally estimate the QoT with better accuracy.

QoT estimation is not, however, the only requirement for network operation, and more powerful tools are needed to support network. In this regard, the digital twin (DT) representation of the optical network was proposed to facilitate network operation [15]. Other DT approaches are also available in the literature. The GN in Python (GNPy) project [16] models the physical layer and shows applications in network planning and operation. The authors in [17] modeled the wavelength-dependent gain characteristics of erbium-doped fiber amplifiers (EDFA) for estimating optical SNR and fiber nonlinearities. The authors in [18] showed the importance of monitoring to improve the accuracy of QoT estimation.

In this paper, we assume the optical time domain OCATA DT [19]. OCATA is able to predict optical signal propagation in the time domain. OCATA models the propagation of in-phase (I) and quadrature (Q) optical constellations along the optical elements in the route of a given lightpath. Applications of the OCATA DT include QoT estimation and failure management for single- and multicarrier signals in the C-band, as well in the C+L+S bands. OCATA has been experimentally demonstrated as a reliable and low-complexity approach for accurately estimating the QoT, specifically the pre-FEC BER, of a lightpath.

With all the above in mind, in this paper, we first focus on analyzing the SSFM and RK4IP signal propagation methods as simulation tools for generating the datasets needed for creating ML models for the MB OCATA DT. Specifically, Section 2 introduces the targeted MB transmission system and overviews the SSFM and RK4IP methods for solving the NLSE modelling optical propagation. In particular, the RK4IP method is proposed as an alternative to the SSFM in the case of MB transmission as it can solve the NLSE faster [20]. In addition, we propose using an adaptive step size algorithm to further reduce the computation time.

Next, Section 3 is devoted to the application of ML for modelling MB optical signal propagation to produce estimate outputs in a faster way. Propagation modelling has usually been carried out by modelling individual optical components, e.g., an optical span, in such a way that component models can be concatenated to model end-to-end lightpaths. In this paper, we evaluate two different approaches: (i) end-to-end lightpath modelling and (ii) per-optical-span modelling. Specifically, two ML techniques are considered: (i) the functional link artificial neural network (FLANN), which is known for its simplicity and ability to model linear and nonlinear effects—because of their characteristics, FLANNs can be used to model end-to-end lightpaths; and (ii) the deep neural network (DNN), which produces very accurate models in the presence of nonlinear effects but is more complex than a FLANN, thus seeming ideal for modelling optical spans. The models are part of the MB OCATA DT architecture, so additional algorithmic solutions are presented, including generation of samples and QoT estimation.

Section 4 presents the results from exhaustive simulation to evaluate the performance of both analytical methods and ML models. The dataset [21] was generated by running RK4IP for several MB optical system configurations. Finally, Section 5 draws the main conclusions of the work.

## 2. Numerical Methods for C+L+S Optical Signal Propagation

**Figure 1:**
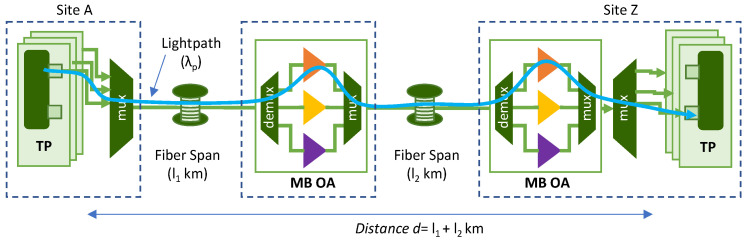
General scenario of C+L+S MB transmission. Illustrative example of a lightpath.

In this section, we consider a C+L+S MB transmission system such as the one illustrated in Figure 1, where a lightpath of total distance *d* connects sites A and Z using channel *λ_p_*. The transponders (TP) include a transmitter (Tx) and a receiver (Rx), and we assume that it may operate in the different transmission bands. The generated signals are multiplexed and transmitted over the optical fiber, where MB optical amplifiers (OA) amplify the optical signal at every span. Two optical spans of lengths *l*_1_ and *l*_2_ can be observed in Figure 1. We consider an MB OA as a system with one OA per band, e.g., an EDFA for the C and L bands and a thulium-doped fiber amplifier (TDFA) for the S band, together with waveband (de)multiplexers.

The propagation of the MB WDM optical signal travelling through the optical fiber can be modelled using the NLSE. In this regard, it is worth noting that channels in each of the C+L+S bands are impacted differently not only by linear effects such as dispersion and fiber attenuation but by the ISRS effect, that potentially increases nonlinearities.

In the next subsections, we first introduce the NLSE and then present the SSFM [6], which is probably the most popular approach for solving the NLSE. In view of the high computational complexity of using the SSFM on fully-loaded MB systems, we propose the RK4IP method [7,8] as an alternative approach for solving the NLSE faster. Finally, an adaptive step size algorithm is presented to further reduce the computation time.

### 2.1. Split-Step Fourier Method

The NLSE is the most accurate tool for modelling the propagation of optical signals along the optical fiber and includes both linear and nonlinear transmission effects [6]. We concentrate on the most relevant effects, such as chromatic dispersion and fiber attenuation linear effects and self-phase modulation and cross-phase modulation nonlinear Kerr-effects. In addition, the energy transferred from incident photons to the vibrational modes results in the generation of new photons with different frequencies, which originates the ISRS and the self-steepening effect.

In this case, the NLSE modelling the signal propagation in the time domain (*t*) can be written as Equation (1) [6,7], where *ω*_0_ is the carrier frequency, coefficients *β*_(·)_ represent the different orders of dispersion, *A*(*z*,*t*) is the field of the optical signal at propagation distance *z*, *R*(*t*) is the nonlinear response function, and *γ* is the nonlinear coefficient.

(1) $$\frac{dA(z,t)}{dz}+\frac{\alpha }{2}A(z,t)+\beta_{1}\frac{dA(z,t)}{dt}+i\beta_{2}\frac{d^{2}A(z,t)}{dt^{2}}-\beta_{3}\frac{d^{3}A\left(z,t\right)}{dt^{3}}\;\;=i\gamma \left(1+\frac{i}{\omega_{0}}\frac{d}{dt}\right)\left(A\left(z,t\right)\int R\left(t^{\prime }\right){\cdot A\left(z,\left(t-t^{\prime }\right)\right)}^{2}{dt}^{\prime }\right)$$

*R*(*t*) can be computed as Equations (2)–(5), where *h_R_*(*t*) is the Raman response function, *f_R_* is the peak Raman gain, and parameters *f*_(·)_ and *τ*_(·)_ adjust the Raman response function.

(2) $$R\left(t\right)=\left(1-f_{R}\right)\delta \left(t\right)+f_{R}h_{R}\left(t\right)$$

(3) $$h_{R}\left(t\right)=\left(f_{a}+f_{c}\right)h_{a}\left(t\right)+f_{b}h_{b}\left(t\right)$$

(4) $${\;h}_{a}\left(t\right)=\tau_{1}\left(\tau_{1}^{-2}+\tau_{2}^{-2}\right)e^{-\left(t/\tau_{2}\right)}\sin\left(t/\tau_{1}\right)$$

(5) $${\;h}_{b}\left(t\right)=\left[{(2\tau }_{b}-t)/\tau_{b}^{2}\right]e^{\left(t/\tau_{b}\right)}$$

In Equation (1), we observe that the amplitude of the optical signal depends on both transmission distance and time. In consequence, two sources of numerical error can be identified when solving the NLSE coming from the size of the related steps. The numerical error coming from the time derivative (which also depends on the distance step size) can be circumvented by rewriting Equation (1) in the frequency domain (*ω*) as:

(6) $$\frac{d\tilde{A}}{dz}+i\cdot \tilde{A}\cdot (\beta \left(\omega \right)-\beta_{0}-\beta_{1}\cdot \omega )\;\;=-i\cdot \gamma \cdot \left(1+\frac{\omega }{\omega_{0}}\right)\;\;\cdot F\left[\left(1-f_{R}\right)\cdot A\cdot {\left|A\right|}^{2}+f_{R}\cdot A{\cdot F}^{-1}\left[{\tilde{h}}_{R}\;\cdot F\left[{\left|A\right|}^{2}\right]\right]\right]$$

where

(7) $$\tilde{A}\left(z,\omega \right)=FT(A(z,t))$$

(8) $${\tilde{h}}_{R}=FT\left(h_{R}\left(t\right)\right)$$

and *FT* denotes the Fourier transform operator.

The split-step Fourier method (SSFM) [6] is the most common method for solving the NLSE. The SSFM relies on computing the solution in small steps while computing the linear and the nonlinear parts separately; the linear part is solved in the frequency domain, whereas the nonlinear part is solved in the time domain.

### 2.2. Fourth-Order Runge–Kutta in the Interaction Picture Method

The RK4IP method can be expressed in the time domain as:

(9) $$A\left(z,t\right)=F^{-1}\left[exp(\frac{h}{2}\cdot D)\cdot F\left[A_{1}+K_{1}/6+K_{2}/3+K_{3}/3\right]\right]+K_{4}/6$$

(10) $$A_{1}=F^{-1}\left[exp(\frac{h}{2}\cdot D)\cdot \tilde{A}(z,t)\right]$$

(11) $$K_{1}=F^{-1}\left[exp(\frac{h}{2}\cdot D)\cdot F\left[h\cdot N(A(z,t))\right]\right]$$

(12) $$K_{2}=h\cdot N\left(A_{1}+\frac{K_{1}}{2}\right)$$

(13) $$K_{3}=h\cdot N\left(A_{1}+\frac{K_{2}}{2}\right)$$

(14) $$K_{4}=F^{-1}\left[h\cdot N(exp\left(\frac{h}{2}\cdot D\right)\cdot F\left[A_{1}+K_{3}\right])\right]$$

where *D* is the dispersion operator, *K*_(·)_ are the RK method coefficients, and *N*(·) is the nonlinear operator.

The RK4IP method can also be applied in the frequency domain, as:

(15) $$\tilde{A}\left(z,\omega \right)=exp(\frac{h}{2}\cdot D)({\tilde{A}}_{1}+{\tilde{K}}_{1}/6+{\tilde{K}}_{2}/3+{\tilde{K}}_{3}/3)+{\tilde{K}}_{4}/6$$

(16) $${\tilde{A}}_{1}=F^{-1}\left[exp(\frac{h}{2}\cdot D)\cdot \tilde{A}(z,\omega )\right]$$

(17) $${\tilde{K}}_{1}=exp\left(\frac{h}{2}\cdot D\right)h\cdot \tilde{N}\left(\tilde{A}\left(z,\omega \right)\right)$$

(18) $${\tilde{K}}_{2}=h\cdot \tilde{N}\left({\tilde{A}}_{1}+\frac{{\tilde{K}}_{1}}{2}\right)$$

(19) $${\tilde{K}}_{3}=h\cdot \tilde{N}\left(\tilde{A}+\frac{{\tilde{K}}_{2}}{2}\right)$$

(20) $${\tilde{K}}_{4}=h\cdot \tilde{N}(exp\left(\frac{h}{2}\cdot D\right){(\tilde{A}}_{1}+{\tilde{K}}_{3}))$$

### 2.3. Adaptive Step Size Algorithm

An adaptive step size algorithm can be defined to further reduce the computation time required by the SSFM and RK4IP methods [22]. Algorithm 1 sketches the pseudocode. The algorithm receives as input the current step size (Δ*z*) and the global error/tolerance (*δ_G_*) and returns the new step size. The algorithm first computes the signal waveform error (*δ*) (line 1 in Algorithm 1) and then updates the step size accordingly (lines 2–4). The algorithm ensures that the step size does not exceed a maximum value specified by *MAX_*Δ*z* (line 5) and returns the new step size (line 6).


**Algorithm 1**. Adaptive step size algorithm for MB WDM signal propagation.**INPUT:** Δ*z, δ_Gf_* **OUTPUT:** Δ*z*1:*δ ← LEM*(·)2:**if** *δ < δ_G_/2* **then** Δ*z_new_ ←* Δ*z* ∗ 2*^η^*3:**else if** *δ > 2 δ_G_* **then** Δ*z_new_ ←* Δ*z*/24:**else** Δ*z_new_ ←* Δ*z*5:**if** Δ*z_new_* > *MAX_*Δ*z*
**then** Δ*z_new_ ← MAX_*Δ*z*6:**return** Δ*z_new_*


We consider the local error method (LEM) [8] approach to estimate *δ* at each step. The LEM estimates the step size at each iteration by using two solutions of Equation (15), the coarse *Ã_c_* (*z*, *ω*) and the fine *Ã_f_* (*z*, *ω*) ones. Solution *Ã_c_* (*z*, *ω*) is calculated over a single full step of size *d*, whereas solution *Ã_f_* (*z*, *ω*) is computed by taking two smaller steps of size *d*/2. It starts with the initial condition *Ã*_1_ and propagates the signal over the first half-step to obtain an intermediate solution *Ã_mid_*. Then, *Ã_mid_* is used as the initial condition to propagate across the second half-step. Finally, the two half-steps are combined to produce the fine solution *Ã_f_* (*z*, *ω*). The local error *δ* is computed as:

(21) $$\delta =\left\|{\tilde{A}}_{f}(z,\omega )-{\tilde{A}}_{c}(z,\omega )\right\|/\left\|{\tilde{A}}_{f}(z,\omega )\right\|$$

## 3. Machine Learning Models

In this section, we focus on modelling optical signal propagation by applying ML techniques to produce estimate outputs quickly. The models can be integrated into the OCATA time domain digital twin, and they can be used for network operation applications, such as QoT estimation during provisioning and failure management.

### 3.1. OCATA MB Time Domain DT Modelling and QoT Estimation

**Figure 2:**
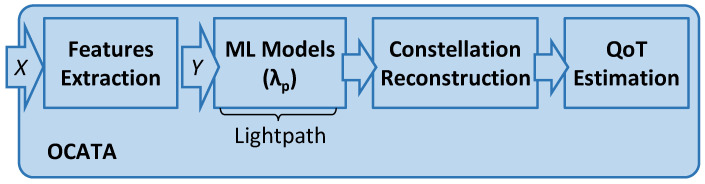
Main building blocks of the OCATA-MB digital twin.

Figure 2 presents the OCATA DT architecture for MB, where incoming optical signals generated using modulation format *m*-QAM are processed in the time domain, i.e., IQ optical constellation samples *X* are used as inputs. Specifically, an input sample *X* consists of a set of symbols x ∈ *X*, where each x = [x^I^, x^Q^] belongs to one of the *m* distinct constellation points (CP). A feature-extraction (FeX) module computes a set of features *Y* characterizing each CP of the received signal by applying Gaussian mixture model (GMM) fitting [23] (see [19] for details). For such characterization, we assume that every CP follows a bivariate Gaussian distribution, so *Y* contains five features for each CP *i*, i.e., *Y^i^* = (*y*)*^i^* = (*μ^I^*, *μ^Q^*, *σ^I^*, *σ^Q^*, *σ^IQ^*)*^i^*, where (1) *μ^I^* and *μ^Q^* represent the mean position for *i* over *I* and *Q* axes, respectively, and (2) *σ^I^* and *σ^Q^* are the variance of *i* over the axes and *σ^IQ^* is the covariance between the axes. As was shown in [19], the dispersion of symbols belonging to each CP provides valuable insight of the level of both linear (LI) and NLI noise affecting the signal, which can be related to QoT-related indicators such as pre-FEC BER and SNR.

Sample generation and FeX can be conveniently combined into a single feature generation module. Algorithm 2 presents such a feature generation module, which produces the initial features to be propagated afterwards. The algorithm receives as input the Tx model; the required Tx configuration, e.g., the modulation format; and the length of the PRBS to be generated. After generating the bit sequence (line 1), the initial IQ optical constellation *X* is obtained by sampling, shaping, and modulating the bit sequence [24] following the received Tx configuration (line 2). Next, GMM fitting is performed to compute the set of bivariate Gaussian distribution that best fits the generated optical constellation *X* (line 3), and the computed features are returned (line 5).


**Algorithm 2**. Feature generator.**INPUT**: *Txmodel*, *Txconfig*, *n_bits***OUTPUT:** *Y*1: 2: 3: 4:*bitSequence* ← PRBS(n_bits) X ← *Txmodel*.generateIQConst (*bitSequence*, *Txconfig*) *Y* ← GMMfitting(*X*) **return** *Y*


The feature generation is then followed by an ML model that estimates the changes in the input features as a result of the propagation of the signal on the optical components in the route of the lightpath. In contrast to the classical OCATA DT for C-band transmission, where ML models are assumed as independent of the specific channel, models need to be trained for the specific channel of interest (*λ_p_*) in the case of MB optical transmission.

In this paper, we evaluated two methodologies for modelling signal propagation: (i) end-to-end lightpath models, which get as inputs features *Y* from the Tx and estimate the features at the Rx after the signal propagates along the route of the lightpath over a distance *d*, and (ii) single optical spans, which include an OA and an optical fiber of a given length. Span models receive a set of input features *Y*, from the signal generated by the Tx or after the signal has traversed some previous optical spans, and produces as output the value of the features characterizing the IQ optical constellation of the optical signal after the propagation through the optical span. In this case, models for end-to-end lightpaths are created by concatenating span models as defined by the route of the lightpath.

Two ML-based approaches were considered: (i) the FLANN, which, because of its simplicity, can be used to model end-to-end lightpaths, and (ii) the DNN, which produces accurate models but, being more complex than FLANNs, is proposed for modelling optical spans.

To reduce the numbers of inputs and outputs, as well as to simplify the structure of the ML models, especially in the case of the DNNs, a subset of selected CPs (*selCP*) was considered for the propagation of the features. Precisely, two exterior and two interior CPs were selected in [19] and, from them, a constellation reconstruction block reconstructed the features for the nonpropagated CPs with high accuracy (Equation (22)). Functions *F^i^* (·) can be defined as linear combinations of the propagated features.

(22) $$\begin{array}{cc} {Y^{i}=F}^{i}\left(\left[Y^{j},\forall j\in selCP\right]\right) & \forall i\in CP \end{array}$$

Finally, the QoT estimation block predicts the QoT of the lightpath from the features of the received signal. Specifically, the parameter *Φ^i^_out_* was defined in [19] as:

(23) $$\Phi_{out}^{i}=1-P\left(x\subset A^{i}|x\sim N\left(Y^{i}\right)\right)$$

where *Φ^i^_out_* represents the probability of receiving a symbol originally sent as part of CP *i* out of the detection area *A^i^* assigned to that CP. Note that the estimated pre-FEC BER can be computed based on *Φ^i^_out_* for all the CPs as Equation (24).

(24) $$\mathrm{pre}-\mathrm{FEC}\;\mathrm{BER}\;\sim \frac{1}{m\cdot \log_{2}\left(m\right)}\sum_{i=1}^{m}\Phi_{out}^{i}$$

In Equation (24), the average *Φ_out_* probability is interpreted as an estimation of the symbol error rate (SER), and the pre-FEC BER is derived assuming that one symbol error causes only one bit with error (which is reasonable under Gray coding).

**Figure 3:**
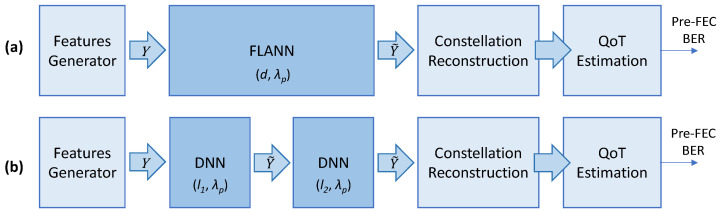
End-to-end (**a**) vs. per-optical-span (**b**) modelling and QoT estimation.

For illustrative purposes, Figure 3 shows the building blocks for the QoT estimation of the lightpath represented in Figure 1 that traverses two optical spans, each of 80 km. In Figure 3a, the FLANN end-to-end model predicts the features of the signal at the receiver in Site Z after the signal propagates on *λ_p_* along *d* km. The constellation reconstruction block estimates the features of the nonpropagated CPs, and the QoT estimation block predicts the pre-FEC BER. In Figure 3b, two span DNN models trained to signal propagation on *λ_p_* along *l*_1_ and *l*_2_ km are concatenated to model the end-to-end lightpath. All the blocks except the models themselves are common for both approaches.

### 3.2. End-to-End Lightpath Modelling Using FLANNs

**Figure 4:**
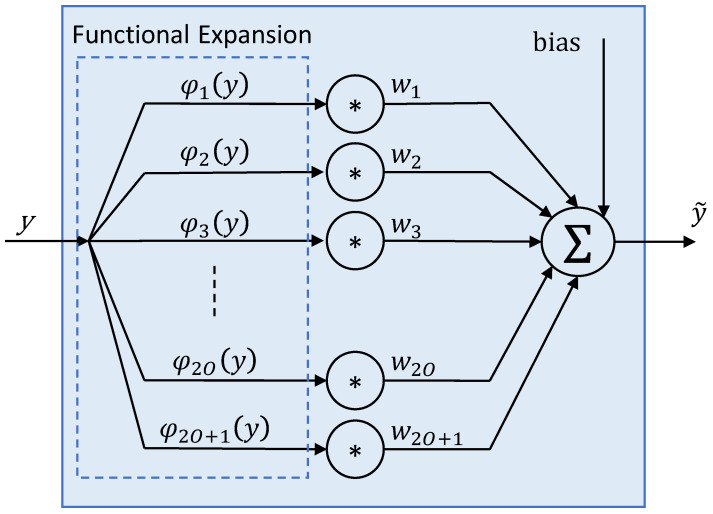
FLANN feature model structure for end-to-end modelling.

The FLANN feature model structure is presented in Figure 4. The complete FLANN model for end-to-end lightpath modelling consists of one feature model for each of the considered features {(*μ^I^*, *μ^Q^*, *σ^I^*, *σ^Q^*, *σ^IQ^*)*^i^*, *i* ∈ *selCP*}.

Each feature model consists of a functional expansion block, where each input feature *y* is extended to capture its nonlinear behavior. In particular, in this paper, we assume that the functional expansion is based on trigonometric functions φ_(·)_(*y*), except φ_1_(*y*) = *y*. Next, each output of the expansion block is multiplied by a weight *w*_(·)_. Because no activation function is used, the estimated value of the feature is given by Equation (25), where *O* is the order of the FLANN model:

(25) $$\tilde{y}=bias+\sum_{k=1}^{2O+1}w_{k}*\varphi_{k}(y)$$

The training of the model aims at finding the weights that minimize the error between the predicted value and the value from the training dataset. FLANN lightpath models can be trained with high precision for each channel and a specific total distance *d* and stored in a model database ready to be used, e.g., during lightpath provisioning.

However, even though FLANN lightpath models can be trained for every channel in the C+L+S bands, they cannot be trained for every arbitrary total distance *d*. A strategy is to pretrain models for incremental distances in steps of the most common span length, e.g., 80 km. However, a real lightpath can traverse different span lengths, e.g., 65 and 75 km, so the models can present imperfections. In addition, variation over time of the behavior of optical devices, e.g., the noise figure of the OAs, new splices in the fiber, etc. can also impact the accuracy of the models. For this very reason, the models should be tuned periodically, using the monitoring measurements collected from the TPs, once the lightpath is established.

### 3.3. DNN-Based Concatenated Model

In this case, span DNNs are specifically trained for every channel and optical span configuration. This reduces the number of models that need to be pretrained as compared with FLANN-based end-to-end lightpath modelling. In addition, NLI noise depends not only on the length of the span but on the accumulated distance that the signal has already traversed at the start of the optical span on the route of the lightpath. In consequence, span DNNs require the accumulated distance *z* as additional input.

**Figure 5:**
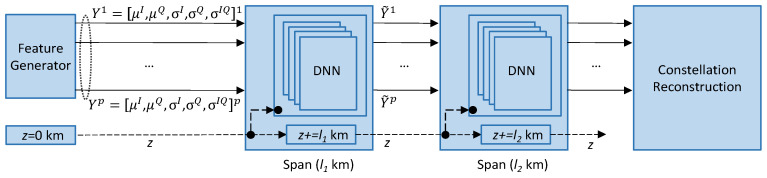
Concatenated DNN model.

The lightpath model is produced by selecting the most appropriate span DNN models from the model database and concatenating them in the order specified by the route of the lightpath. In addition, the traversed distance needs also to be propagated and connected to the related input of the span DNNs. Figure 5 represents the concatenated model for the lightpath represented in Figure 1.

As in the case of the FLANN model, the concatenated DNN model can present imperfections due to the accumulated error and variation of the behavior of optical devices over time. For this very reason, the concatenated DNN models need to be tuned periodically from the monitoring measurements collected from the TPs.

## 4. Results

In this section, we first describe the MB optical system under simulation and modelling. Next, we evaluate the performance of the RK4IP method with adaptive step size w.r.t. the SFFM method in terms of both accuracy and computation time. The selected simulation method was used to generate a large dataset to be used for ML modelling of MB optical propagation, and ultimately, estimating the QoT. The evaluation was carried out on three different channels in each of the bands.

### 4.1. MB Optical System

We assumed that the optical transponders generated 16QAM@32GBd signals shaped by a root-raised cosine filter with a 0.06 roll-off factor. For the C+L+S system, we considered 337 optical channels, with 50 GHz channel spacing for full spectrum usage. Table 1 summarizes the configurations used in the MB transmission simulator.

Pseudorandom binary sequences were used as input for every channel. The signal was propagated through spans of standard single-mode fiber (SSMF), ranging from 70 to 100 km, with a launch power of 0 dBm. Gain-flattening filters were used at the end of each fiber span to compensate for the power tilt induced by the ISRS effect. The attenuation factor, chromatic dispersion, and nonlinear coefficient (*γ*) varied with frequency [25]. The fractional contribution of the delayed Raman response was 0.245 [7].

Propagation in optical spans was modeled by solving the NLSE. In line with [6,7], we considered: *τ*_1_ = 12.2 × 10^−3^ [ps], *τ*_1_ = 32 × 10^−3^ [ps], *τ_b_* = 96·× 10^−3^ [ps], *f_a_* = 0.75, *f_b_* = 0.21, *f_c_* = 0.04, and *f_R_* = 0.18. EDFAs and TDFAs were modeled as ideal OAs characterized by a single gain and noise figures. The adaptive step size algorithm was as defined in Section 2. C, to reduce the computation time, was run with *δ_G_* = 10^−4^, *η* = 5, and *MAX_*Δ*z* = 10 m. Finally, at the Rx, a DSP block performed ideal chromatic dispersion compensation and phase recovery.

### 4.2. B. B. MB Optical System Simulation

The MB system described in the previous section was simulated using a simulator of a coherent WDM system based on MATLAB R2024a. The simulator was executed on an Intel Core i7-6700 processor with 16 GB RAM and running Windows 11 64-bit. For this very reason, pseudorandom sequences were limited to 2^14^ bits in length.

We compared the traditional SSFM method with the RK4IP method enhanced with the adaptive step size algorithm (see Section 2). In addition, a fixed (2 m) step size approach was also considered for comparison purposes. Specifically, four RK4IP methods were evaluated: (1) RK4IP with adaptive step size in the time domain (A-RK_t); (2) RK4IP with adaptive step size in the frequency domain (A-RK_ω); (3) RK4IP with fixed step size in the time domain (F-RK_t); and (4) RK4IP with fixed step size in the frequency domain (F-RK_ω). For the evaluation, we considered one single optical span of 80 km and all the channels with launch power of 0 dBm, i.e., a total launch power of 25.27 dBm. In addition, the SSFM method was executed with a fixed step size of 0.01 m.

The comparison was carried out by comparing the ISRS gain S(*λ_i_*) computed by the RK4IP methods and SSFM for every channel, where S(*λ_i_*) is defined as the ratio between the channel power after fiber propagation and its value without the SRS, i.e., considering *f_R_* = 0 (Equation (26)) [26]. This approach allowed specifically analyzing the feasibility of using the RK4IP methods for MB optical transmission. Comparison of the ISRS gain per channel was averaged using the relative squared error (RSE), defined in Equation (27).

(26) $$S\left(\lambda_{i}\right)=\frac{P(\lambda_{i})}{P_{no\;ISRS}(\lambda_{i})}$$

(27) $$RSE=\frac{1}{N}\sum_{i=1}^{N}\frac{{\left|S_{RK4IP}\left(\lambda_{i}\right)-S_{SSFM}(\lambda_{i})\right|}^{2}}{{\left|S_{SSFM}(\lambda_{i})\right|}^{2}}$$

**Figure 6:**
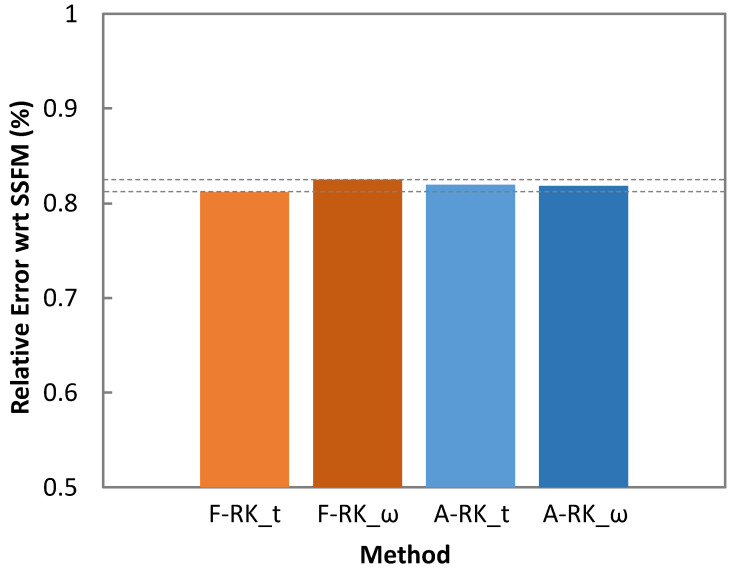
Relative error of RK4IP methods with respect to SSFM.

Figure 6 plots the RSE. All the considered RK4IP methods provided solutions leading to very small error compared with the SSFM method, which makes them valid approaches for simulation of MB optical transmission.

**Figure 7:**
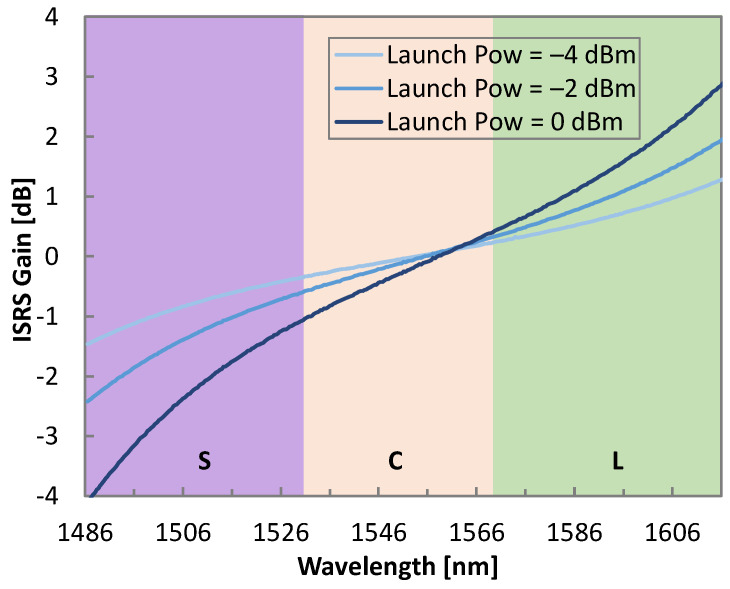
ISRS gain observed for different channel launch powers.

Figure 7 shows the ISRS gain profile after fiber propagation by executing the A-RK_ω method for launch powers of −4, −2, and 0 dBm per channel, i.e., 21.27, 23.27, and 25.27 dBm of total launch power, respectively. A decrease in the ISRS gain tilt from around 7 to just 3 dB was observed as the total input power decreased. Note that even for launch powers as low as −4 dBm per-channel, the impact of ISRS was still noticeable.

**Figure 8:**
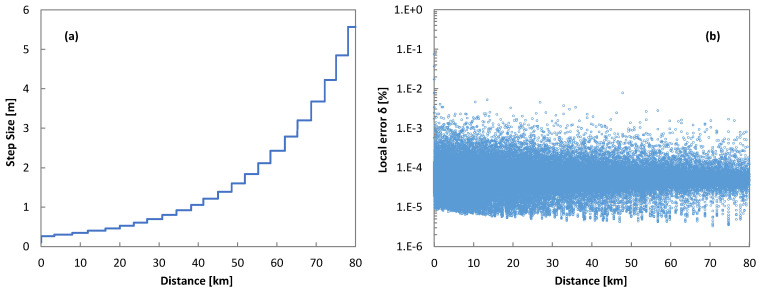
Evolution of the step size (**a**) and error (**b**) with propagation distance.

Figure 8a plots the adaptation of the step size, as provided by Algorithm 1, and Figure 8b plots the error, computed as Equation (21), with the propagated distance. We observe that the adaptive step size algorithm provided good accuracy by fixing small step sizes during the initial propagation and then relaxing the step size when the propagated distance increased.

**Figure 9:**
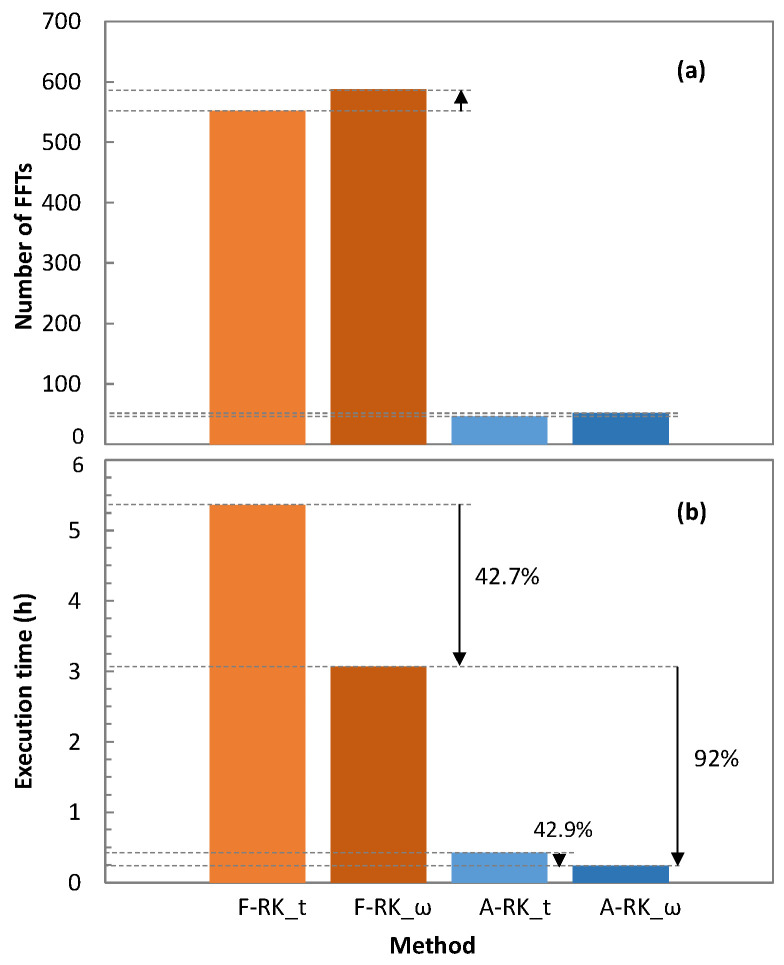
Number of FFTs required (**a**) and total computation time (**b**) of RK4IP methods.

The adaptation of the step size was responsible for a reduction in the number of FFT operations that are needed. Figure 9a reports the number of FFT operations executed during the execution of the different RK4IP methods. We observe that the number of FFT operations heavily decreased when the adaptive step size algorithm was used. This translated into a reduction in the total computation time, as observed in Figure 9b. In particular, the reduction in computation time enabled by the adaptive step size algorithm was over 90% (from over 3 h to just 15 min). Interestingly, running the RK4IP methods in the frequency domain reduced the computation time by over 40% even though the number of FFTs operations was slightly higher than in the time domain. This was a consequence of the higher complexity of the related FFT operations.

In conclusion, the RK4IP with adaptive step size in the frequency domain (A-RK_ω) provided good accuracy when compared with the SSFM method while requiring much smaller computation time. Consequently, we used this method to generate the large dataset required for ML training.

### 4.3. C. C. MB Optical Propagation Models

A dataset [21] with 2^16^ 16-QAM symbols for each of the 337 channels in the optical MB system and for different lightpath distances ranging from 1 to 10 optical spans of 80 km, i.e., from 80 to 800 km, was generated using the A-RK_ω method, as proposed in the previous subsection. Armed with this dataset, FLANN models for end-to-end lightpaths and DNN models for optical spans were trained, validated, and tested.

The FLANN models had (i) 20 inputs for the features of the selected CPs, (ii) order *O* = 2, and (iii) 20 outputs for the propagated features of the lightpath, and (iv) they were trained using the least mean squares (LMS) optimizer with a learning rate of 8 × 10^−3^ and mean squared error as a loss function for up to 1000 epochs with a batch size of 64.

The DNN link models had (i) 21 inputs, i.e., one additional input for accumulated distance, (ii) two hidden layers with 12 neurons and a *tanh*(·) activation function, and (iii) 20 outputs for the propagated features for the optical span, and (iv) they were trained with Adam as an optimization algorithm, with a learning rate of 10^−3^ and mean squared error as a loss function for up to 1000 epochs with a batch size of 64.

To simplify the evaluation, we concentrated on analyzing the performance for one single channel per optical band, specifically channels 1, 167, and 337 in the S, C, and L bands, respectively.

**Figure 10:**
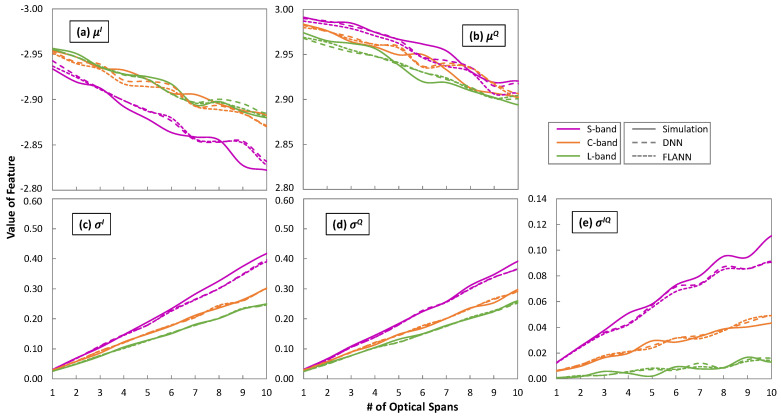
Evolution of the values of the features with the number of optical spans.

**Figure 11:**
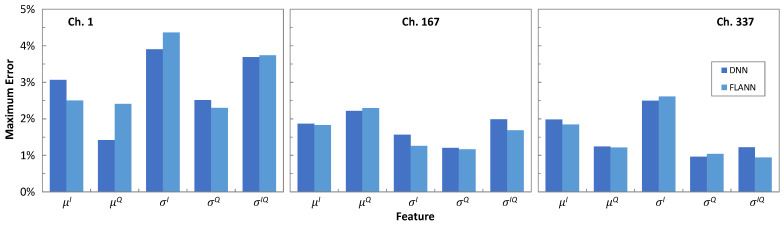
Maximum feature errors of ML models.

Figure 10 plots the evolution of the features *μ^I^*, *μ^Q^*, *σ^I^*, *σ^Q^*, and *σ^IQ^* characterizing the bivariate Gaussian distribution of the exterior CP [−3 + 3i], with the propagation distance in terms of optical spans as obtained from the simulator and predicted by the ML models. In general, the values of the features changed almost linearly with the distance, which was likely due to the simplicity of the trained ML models, i.e., order 2 for the FLANNs and two hidden layers with 12 neurons for the DNNs. With these very simple structures, the predicted features were very close to the ones from simulation. Figure 11 shows the maximum absolute error between the features predicted by the ML models w.r.t. the ones from simulation for CP [−3 + 3i]. In all cases, the error was smaller than 5%.

Among the different impacts on the shape of the CP caused by the evolution of the features observed in Figure 10, the increase in the CP variance was considered the most important, as the increased variance could make some of the received symbols fall out of the detection area assigned to that CP, which would increase the pre-FEC BER. Finally, we observed very different trends for the channels under analysis in the different bands, which also anticipated different optical performance of the associated lightpaths in terms of QoT.

### 4.4. QoT Estimation

**Figure 12:**
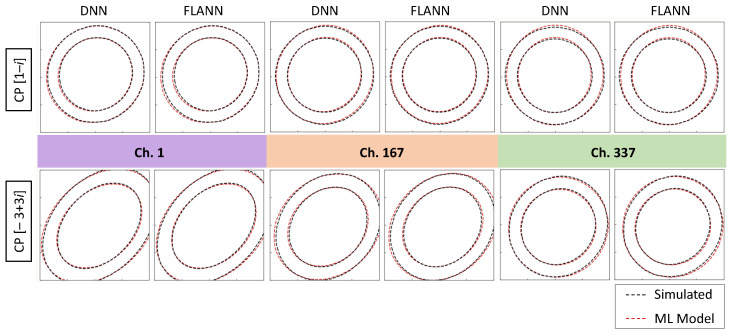
Contours of two CPs from the simulator and ML models.

Before analyzing the accuracy of the QoT estimation, let us observe the shape of two CPs, the interior [1 − *i*] and exterior [−3 + 3*i*], for the three channels under analysis. Figure 12 reproduces the contours representing the different levels of the bivariate Gaussian distributions that characterized the CPs after three optical spans. The contours correspond to levels 0.1 and 0.01 of the variances of the corresponding Gaussian distributions. We observe strong similarities between both distributions for the three different bands, which validates the proposed modelling approaches. It is interesting to observe the changes in the shape of the constellations for the different bands, which are more noticeable in the exterior CP. In particular, we observe that the contours clearly exceeded the detection area for that CP in the case of ch. 1, which is a clear indication of high pre-FEC BER.

With the above in mind, let us evaluate the accuracy of the QoT estimation for the three channels under analysis. We assume a pre-FEC BER threshold equal to 10^–2^ in all the cases. QoT estimation was carried out with the features of all the CPs in the IQ constellation, i.e., after constellation reconstruction.

**Figure 13:**
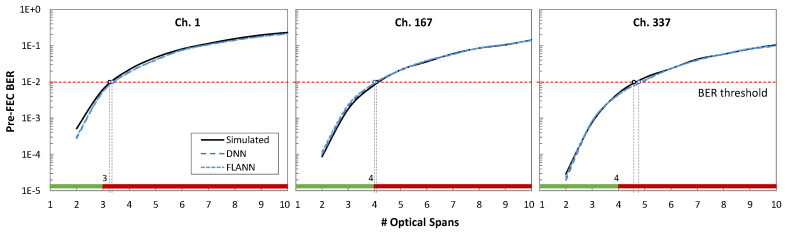
Estimated pre-FEC BER.

Figure 13 plots the pre-FEC BER as a function of the span count for the three channels under analysis. The results show that the models could closely predict the actual QoT for all the channels. We observe remarkably high accuracy in both ML models for QoT estimation. In particular, both models can be used to anticipate whether a candidate route and wavelength assignment will meet the required QoT. Note the importance of wavelength selection for the different channels under analysis, where a lightpath assigned with channel 1 was able to provide pre-FEC BER values only under the threshold of three or fewer spans, while channels 167 and 337 could support one more span.

## 5. Concluding Remarks

The foreseen introduction of MB optical transmission in transport networks requires the availability of accurate and low-complexity tools for network planning and operation. In particular, the introduction of digital twinning solutions can provide the needed support for network automation. In this paper, we first analyzed the feasibility of using SSFM solving of the NLSE for modelling optical signal propagation. We observed that the large number of channels in a fully loaded MB system and the need to consider the ISRS effect discourage using that solution in general, even for generating offline simulation data. In view of that, the RK4IP method was evaluated as an alternative solution to reduce the computation time. The RK4IP method can be solved in the time or frequency domain, so both formulations were considered. In addition, an adaptive step size algorithm to further reduce the computation time is proposed.

For evaluation purposes, four RK4IP methods were compared, considering adaptive and fixed step sizes in the time and frequency domains. The obtained results showed that all four methods provided relative errors well under 1% w.r.t. the SSFM method, with the adaptive step size RK4IP method solved in the frequency domain (A-RK_ω) providing the smallest computation time as a result of the lowest number of FFT operations needed. Therefore, we selected the A-RK_ω method for simulation of MB optical transmission systems with several configurations. The resulting dataset can be used for modelling MB systems using ML, so applications such as estimation of the QoT of unestablished lightpaths can be made available, offering high accuracy with very low complexity.

In this paper, we target modelling the signal propagation in the time domain, so the models can be integrated into the OCATA optical time domain DT. Two modelling approaches are proposed and evaluated in this paper. The first approach targets modelling the propagation of the optical signal along an end-to-end lightpath, while the second targets modelling single optical spans so models of end-to-end lightpaths can be easily created by concatenating span models as defined by the route of the lightpath. In both cases, different models were pretrained using the dataset generated by simulation, thus producing a model dataset where each model is characterized by the length of the lightpath or the optical span and the used channel. Two ML techniques were investigated: (i) FLANNs, which, because of their simplicity, are used for modelling end-to-end lightpaths, and (ii) DNNs, which are proposed for modelling single optical lightpaths. In both cases, the models were integrated into OCATA, so tools for features generation, constellation reconstruction, and QoT estimation were used.

The ML modelling approaches were evaluated in terms of their accuracy for modelling features propagation, as well as in terms of the accuracy of their QoT estimation, specifically of the pre-FEC BER. The results showed that the accuracy of the approaches was very similar and very high in general, with error well below 5% w.r.t. to the data from simulation. Regarding pre-FEC BER estimation, the results showed not only the ML models’ accuracy in estimating the pre-FEC BER value but their application for candidate route and wavelength assignment validation by comparing the estimates to the BER threshold.

In conclusion, both approaches were validated to be integrated in the OCATA DT. Even though optical span modelling seems more flexible, as it reduces the number of models to be pretrained, the availability of both approaches provides a method for checking deviations in the models and adds robustness to OCATA.

## Figures and Tables

**Table 1:** Considered MB optical systems.

	S Band	C Band	L Band
Wavelength range [nm]	1486.5–1530.0	1530.4–1568.4	1568.8–1621.5
Bandwidth [THz]	5.7	4.75	6.2
Num. of channels (Ids)	116 (1–116)	96 (117–212)	125 (213–337)
Type of amplifier	TDFA	EDFA	EDFA
Amplifier noise figure [dB]	6.5	5	5
Nonlinear coefficient range [(W m)^–1^]	1.31–1.41	1.24–1.31	1.13–1.23
Dispersion range [ps/nm/km]	12.55–15.58	15.60–18.02	18.1–21.69
Attenuation range (dB/km)	0.19–0.21	0.18–0.19	0.18–0.20

## Data Availability

Data are available in [21].
